# Surgical treatment for bacterial meningitis after spinal surgery: A case report

**DOI:** 10.1097/MD.0000000000006099

**Published:** 2017-03-24

**Authors:** Li-Min Zhang, Liang Ren, Zhen-Qi Zhao, Yan-Rui Zhao, Yin-Feng Zheng, Jun-Lin Zhou

**Affiliations:** a Department of Orthopedics, Beijing Chao-Yang Hospital, Capital Medical University, Beijing; b Department of Spinal Surgery, The Third Hospital of Shijiazhuang, Shijiazhuang, People's Republic of China.

**Keywords:** cerebrospinal fluid leakage (CSFL), postoperative meningitis, pseudomeningocele, spinal surgery

## Abstract

**Rationale:**

Bacterial meningitis (BM) has been recognized as a rare complication of spinal surgery. However, there are few reports on the management of postoperative BM in patients who have undergone spinal surgery. The initial approach to the treatment of patients suspected with acute BM depends on the stage at which the syndrome is recognized, the speed of the diagnostic evaluation, and the need for antimicrobial and adjunctive therapy.

**Patient concerns:**

Here, we report the case of a patient with lumbar spinal stenosis and underwent a transforaminal lumbar interbody fusion at L4–L5. The dura mater was damaged intraoperatively. After the surgery, the patient displayed dizziness and vomiting. A CSF culture revealed *Pseudomonas aeruginosa* infection.

**Diagnoses:**

The patient was diagnosed with postoperative BM.

**Interventions:**

Antibiotic was administered intravenously depends on the organism isolated. Nevertheless, the patient's clinical condition continued to deteriorate. The patient underwent 2 open revision surgeries for dural lacerations and cyst debridement repair.

**Outcomes:**

The patient's mental status returned to normal and her headaches diminished. The patient did not have fever and the infection healed.

**Lessons:**

Surgical intervention is an effective method to treat BM after spinal operation in cases where conservative treatments have failed. Further, early surgical repair of dural lacerations and cyst debridement can be a treatment option for selected BM patients with complications including pseudomeningocele, wound infection, or cerebrospinal fluid leakage.

## 1 Introduction

The occurrence of bacterial meningitis (BM) as a complication of spinal surgery, which is usually the result of a direct bacterial invasion of the meninges through a dural rupture with cerebrospinal fluid (CSF) leakage, is seldom reported.^[1]^ However, a spinal CSF fistula may develop into BM, which occurs when the extradural CSF communicates with another cavity or when direct contact with the outside exists.

CSF analysis and culture remain the definitive method for the diagnosis of meningitis.^[2]^ However, early differentiation of postoperative BM from postoperative aseptic meningitis (PAM) is difficult. This differentiation has been reported to depend exclusively on CSF culture results.^[3]^ CSF culture is highly specific; however, it lacks sensitivity, especially when antimicrobials have previously been administered. The time needed for results to appear is a further limitation of this method.^[4]^ The initial approach to the treatment of patients suspected with acute BM depends on the stage at which the syndrome is recognized, the speed of the diagnostic evaluation, and the need for antimicrobial and adjunctive therapy.^[5,6]^

Here, we describe the case of a patient who developed postoperative BM after surgery for lumbar spinal stenosis. The patient was free from meningitis after surgical intervention for dural lacerations and cyst debridement repair.

## 2 Case report

This study was approved by the Research Ethics Committee of The Third Hospital of Shijiazhuang, Shijiazhuang, China. The patient provided written informed consent for the publication of the present case report.

A 58-year-old Chinese woman was admitted to our institution with progressive neurogenic claudication unresponsive to conservative treatment. She had rheumatoid arthritis, which for 18 years had been poorly controlled with methylprednisolone and leflunomide treatment.

**Figure 1:**
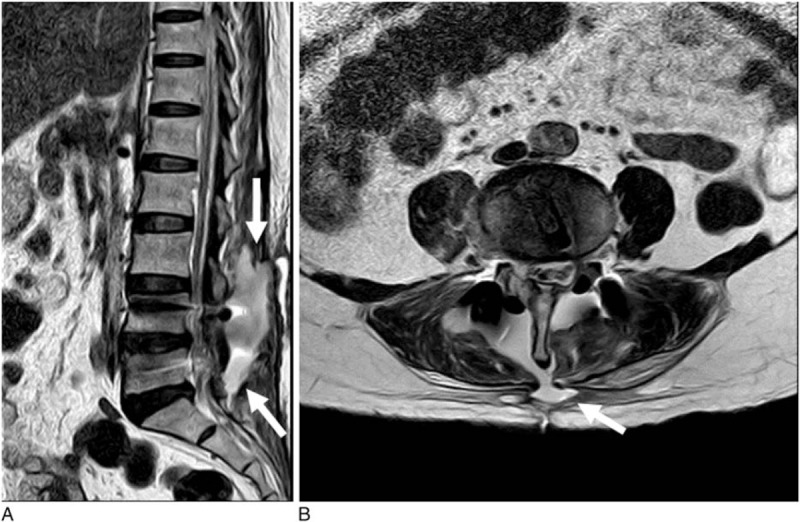
Lumbosacral MRI performed on the 18th day after posterior lumbar and interbody fusion at L4–L5. (A) Sagittal T2-weighted image showing a large amount of cerebrospinal fluid (arrows) in the lumbosacral space. (B) Axial T2-weighted MRI showing a cerebrospinal fluid cutaneous fistula (arrow). MRI = magnetic resonance imaging.

The patient was diagnosed with lumbar spinal stenosis and underwent a transforaminal lumbar interbody fusion at L4–L5. The dura mater was damaged intraoperatively; however, it was not sutured owing to the size of the dural tear (5 mm) and its location (lateral gutter). Postoperatively, the patient was prescribed 10 days of bed rest in the supine position. On the 16th postoperative day, a bulging mass with a ballotable collection of fluid was detected under the wound, and 60 mL of turbid CSF were withdrawn through a wound puncture. A culture of the CSF revealed penicillin-sensitive *Enterococcus faecalis*. Lumbosacral magnetic resonance imaging (MRI) performed on the 18th postoperative day demonstrated a large amount of CSF leakage in the lumbosacral space (Fig. 1).

**Figure 2:**
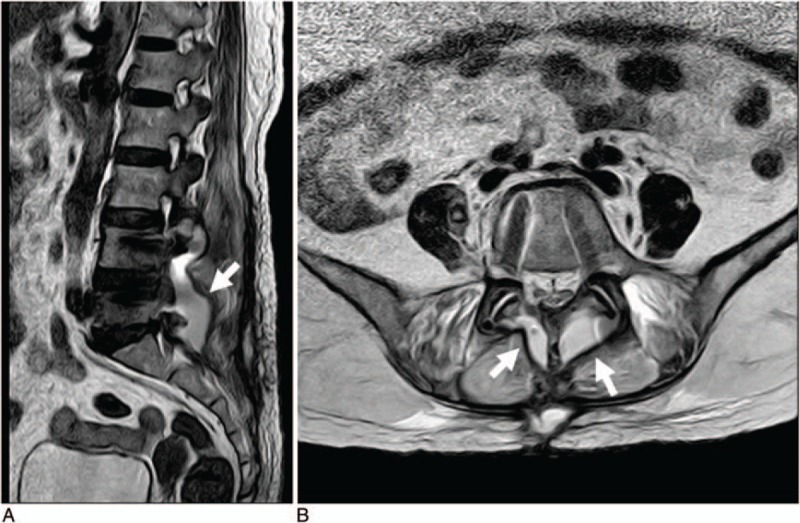
On the 19th day after the second operation, a large amount of cerebrospinal fluid leakage was noted on T2-weighted MRI. (A) Sagittal T2-weighted image showing a large amount of cerebrospinal fluid leakage in the lumbosacral space (arrow). (B) Axial T2-weighted MRI showing a large amount of cerebrospinal fluid leakage (arrows). MRI = magnetic resonance imaging.

The patient underwent the second operation to repair the torn dura mater and debride the cyst on the 19th postoperative day. During the surgery, a dural defect was noted on the L5 thecal sac. The dural defect was sutured with a 5-0 Prolene suture and a muscle plug. She received intravenous administration of penicillin (8 million units twice daily) from the first to the fourth day after the second surgery. The patient presented with pyrexia (38.4°C) and severe headaches after 3 days of the second surgery. Cutaneous CSF fistula was diagnosed by inspection of the surgical wound. The peripheral white blood cell count was 10.24 × 10^9^/L, the erythrocyte sedimentation rate was 47 mm/h, and the C-reactive protein level was 36.3 mg/dL. To treat this complication, vancomycin (1 g twice daily) was administered instead of penicillin. Eight days after the second surgery, a lumbar puncture and a continuous lumbar subarachnoid drainage were performed after an emergent brain computed tomography scan, which ruled out any space-occupying lesions within the brain parenchyma. However, the lumbar subarachnoid drainage tube was blocked after 1 day of its placement. Ten days after the second surgery, the patient displayed dizziness and vomiting. Additionally, Kernig's sign was observed. CSF withdrawn via lumbar subarachnoid drainage showed a leukocyte count of 46/mm^3^, a protein concentration of 1.05 g/L, and a glucose concentration of 5.19 mmol/L. A CSF culture revealed *Pseudomonas aeruginosa*, which was sensitive to vancomycin, meropenem, piperacillin, and tazobactam. The patient was then diagnosed with postoperative BM. Piperacillin and tazobactam (4.5 g twice daily for 8 days) were administered intravenously to treat this episode of *P aeruginosa* infection. Nevertheless, the patient's postoperative clinical condition continued to deteriorate. On the 19th day after the second surgery, a large amount of CSF leakage was noted on T2-weighted MRI (Fig. 2).

On the next day (20th postoperative day), debridement was repeatedly performed and the laceration of the dura matter was repaired with a fascial graft and fibrin glue. Further, an epidural drain was placed, and the paraspinal muscles and fascia were closed in 2 layers, in a figure-of-eight fashion, to create a watertight closure. Treatment with vancomycin (0.5 g twice daily for 17 days) and meropenem (0.5 g twice daily for 20 days) was started after the third operation. The patient improved rapidly and was discharged home after 20 days of intravenous antibiotic administration. However, 9 days after discharge, she was readmitted because of the reappearance of severe headaches and an altered mental status. A lumbar puncture revealed a purulent CSF in addition to a CSF leukocyte count of 2560/mm^3^, a protein concentration of 1.95 g/L, and a glucose concentration of 2.87 mmol/L. A culture of the CSF revealed an *Escherichia coli* infection. Meropenem (500 mg) was administered intravenously, twice a day for 14 days. After 3 days of antibiotic therapy, her mental status returned to normal and her headaches diminished. The patient did not have fever thereafter and the infection healed. Thus, she was discharged from the hospital on the 20th day of hospitalization. At a 1-year follow-up visit, the patient reported not having any headaches and she continued to live independently. The infection was cured without any sequelae.

## 3 Discussion

BM is a severe, potentially life-threatening infection that is associated with high rates of morbidity and significant disability among survivors.^[6]^ Even with modern therapies, the mortality rate of pneumococcal meningitis patients, the most prevalent BM subgroup, reaches 30%.^[7]^ Lin et al^[8]^ reported that out of 20,178 patients who underwent spinal operations in their institution, 21 (0.10%) were diagnosed with postoperative meningitis. Twyman et al^[9]^ described 4 cases of BM that were identified after 2180 operations, an incidence of 0.18%. It is difficult to determine the true incidence of postoperative meningitis following incidental durotomies because many cases are asymptomatic.

The occurrence of BM, as a complication of spinal surgery, usually resulting from direct bacterial invasion of the meninges through a dural rupture with CSF leakage, is seldom reported. The initial approach to the treatment of patients with suspected acute BM depends on the stage at which the syndrome is recognized, the speed of the diagnostic evaluation, and the need for emergent antimicrobial and adjunctive therapy.^[10]^ CSF culture is presently a reliable method for the diagnosis of BM. However, early differentiation of postoperative BM from PAM is very difficult. This distinction is important because BM requires urgent intravenous antibiotic administration, whereas PAM is self-limiting.^[11]^ The present capacity to differentiate between postoperative BM and PAM has been reported to depend exclusively on CSF culture results.^[3]^ However, CSF culture lacks sensitivity, especially in cases of previous antibiotic use.^[4,6,12]^ A systematic review by Huy and colleagues summarizes data from 25 studies evaluating the role of CSF lactate in the differential diagnosis between acute bacterial and aseptic meningitis. The authors concluded that CSF lactate is a good single indicator and a better marker compared with conventional markers.^[13]^ In our case, the patient was diagnosed with BM on the basis of her clinical presentations and CSF culture.

The quality of the existing evidence regarding therapies for postoperative BM is insufficient, and there is a need for further studies. The first treatment for BM is antibiotic therapy. Postoperative meningitis can be caused by staphylococci and aerobic Gram-negative bacilli (including *P aeruginosa*).^[12,14]^ Therefore, vancomycin in addition to either cefepime, ceftazidime, or meropenem is recommended as empirical antimicrobial therapy for adult patients with BM post-surgery.^[5,12]^ Yoshiharu had reported a case of postoperative meningitis treated with linezolid, with a satisfactory clinical outcome after 16 days treatment.^[15]^ However, antibiotics administered through the intravenous or even lumbar intrathecal route do not always enter the intracranial CSF-containing spaces. The reasons for this are uncertain; however, they probably include failure of drug transport to the CSF, dilution of the drug by the CSF, and occlusion of the Sylvian aqueduct or outlets of the fourth ventricle by inspissated pus.^[16]^ Antibiotic resistance is another important consideration.^[17]^

Additionally, surgery may be a useful adjunct in certain cases. Lin et al retrospectively reviewed 21 patients diagnosed with postoperative meningitis after lumbar spinal surgery. All 21 patients survived and recovered completely after at least 2 weeks of antibiotic treatment. However, pseudomeningocele and poor wound healing manifested as complications in 3 patients, in whom further surgery for dural repair was performed.^[8]^ Thus, surgical treatment repair of dural lacerations and cyst debridement have an advantage in the treatment of meningitis complicated with pseudomeningocele, wound infection, or CSF leakage. It is important to repair all dural tears and to administer antibiotics that cover uncommon bacteria to those who develop symptoms of meningitis.^[18]^ The occurrence of pseudomeningocele is a possible sequela of a dural tear. It has been reported to be related to imperfect suture of the dura or fascia, and it may require surgical intervention.^[8,19,20]^ In the present case, inappropriate antibiotic administration and imperfect suture of the dura or fascia during treatment may have contributed to the initial development of meningitis, and the meningitis relapse may have occurred owing to the short-term antibiotic treatment. Nevertheless, the patient was successfully cured of recurrent meningitis and did not develop side effects.

## 4 Conclusions

In conclusion, this case report shows that surgical intervention is an effective method to treat BM after spinal operation in cases where conservative treatments have failed. Furthermore, early surgical repair of dural lacerations and cyst debridement can be a treatment option for selected patients with BM complicated with pseudomeningocele, wound infection, or CSF leakage. It is important to consider the possibility of BM in any patient with CSF leakage after spinal surgery. We recommend the use of CSF cultures and diagnostic imaging as early as possible to ensure prompt diagnosis and treatment.
